# All-cause and cause-specific mortality risk among men and women with hepatitis C virus infection

**DOI:** 10.1371/journal.pone.0309819

**Published:** 2024-09-09

**Authors:** Hung-Wei Wang, Yen-Chung Wang, Yun-Ting Huang, Ming-Yan Jiang

**Affiliations:** 1 Division of Nephrology, Department of Internal Medicine, Chi Mei Hospital Chiali, Tainan, Taiwan; 2 Division of Hepato-Gastroenterology, Department of Internal Medicine, Chi Mei Hospital Liouying, Tainan, Taiwan; 3 Division of Nephrology, Department of Internal Medicine, Chi Mei Medical Center, Tainan, Taiwan; 4 Department of Pharmacy, Chia Nan University of Pharmacy & Science, Tainan, Taiwan; Kaohsiung Medical University, TAIWAN

## Abstract

**Background:**

Hepatitis C virus (HCV) infection affects men and women differently, yet few studies have investigated sex differences in long-term mortality risk among the HCV-infected population. We conducted a population-based study to elucidate all-cause and cause-specific mortality among men and women with HCV infection.

**Methods:**

The study population consisted of adult participants from the 1999–2018 National Health and Nutrition Examination Survey, including 945 HCV-infected and 44,637 non-HCV-infected individuals. HCV infection was defined as either HCV seropositivity or detectable HCV RNA. Participants were followed until the date of death or December 31, 2019, to determine survival status.

**Results:**

The HCV-infected population, both male and female, tended to be older, more likely to be Black, single, have lower income, lower BMI, higher prevalence of hypertension, and were more likely to be current smokers. During a median follow-up of 125.0 months, a total of 5,309 participants died, including 1,253 deaths from cardiovascular disease (CVD) and 1,319 deaths from cancer. The crude analysis showed that the risk of death from all causes and from cancer, but not from CVD, was higher in the HCV-infected population. After adjusting for potential confounders, we found that both HCV-infected men (HR 1.41, 95% CI 1.10–1.81) and women (HR 2.03, 95% CI 1.36–3.02) were equally at increased risk of all-cause mortality compared to their non-HCV infected counterparts (p for interaction > 0.05). The risk of cancer-related mortality was significantly increased in HCV-infected women (HR 2.14, 95% CI 1.01–4.53), but not in men, compared to non-HCV-infected counterparts. Among HCV-infected population, there was no difference in the risks of all-cause, CVD-related, or cancer-related death between men and women.

**Conclusion:**

Both men and women with HCV infection had an increased risk of death from all causes compared to their non-HCV infected counterparts, but we did not observe a significant sex difference.

## Introduction

Hepatitis C virus (HCV) infection is prevalent worldwide and constitutes a significant public health burden [1]. As of 2015, the global prevalence of chronic HCV infection was estimated at 1.0%, affecting approximately 71 million people [1]. In the United States (U.S.), more than 2 million adults are estimated to be HCV-infected [2, 3], with a prevalence of 1.3% among males and 0.6% among females [4]. Healthcare utilization is higher among HCV-infected individuals [5]. In addition to chronic liver disease, cirrhosis, and hepatocellular carcinoma, HCV infection is also associated with several extrahepatic diseases such as cardiovascular diseases, kidney disease, and non-liver related malignancies [6–8].

HCV infection affects men and women differently. In Western countries, injection drug use is a major risk factor for acquiring HCV infection [9, 10]. Women who inject drugs are more likely than men to contract the infection [11], yet HCV-infected women often exhibit a more favorable virus clearance rate compared to men [12, 13]. Moreover, the progression of HCV infection tends to be worse in men than in women [13–15]. However, the risk of fibrosis in women changes over time and is directly influenced by reproductive status, as estradiol and estrogen receptors in the liver protect hepatocytes from oxidative stress, inflammatory injury, and cell death [13].

Despite these differences, there is limited research investigating mortality risk disparities between HCV-infected men and women. Therefore, we conducted a population-based cohort study to elucidate the risk of all-cause and cause-specific mortality among men and women with HCV infection.

## Methods

### Data source

Our data were sourced from the U.S. National Health and Nutrition Examination Survey (NHANES), which employs a series of cross-sectional, multistage probability sampling methods to survey the civilian noninstitutionalized population nationwide (https://wwwn.cdc.gov/nchs/nhanes/tutorials/module2.aspx). NHANES collects data in 2-year cycles through health-related questionnaires, physical examinations, and laboratory tests. The data are publicly accessible on the National Center for Health Statistics website (https://www.cdc.gov/nchs/nhanes/index.htm.). NHANES protocols were approved by the research ethics review board of the National Center for Health Statistics, with all the participants providing written informed consent.

### Study population

In this retrospective cohort study, we combined the data from 10 discrete 2-year cycles of the continuous NHANES, spanning from 1999–2000 to 2015–2016, to create our study population (n = 101,316). We excluded individuals aged younger than 19 years (n = 46,235) or older than 80 years (n = 4,257) at the time of examination, those who did not undergo HCV testing (n = 5,155), and whose mortality status was unavailable (n = 87). Finally, our analysis included 45,582 participants aged 20 to 79 years. The data were accessed on 27 August 2023. Information about individual participants could not be identified during data collection.

### Exposure

The primary independent variable was the HCV infection status, defined by the results of HCV antibody tests and HCV RNA tests. The HCV antibody test was performed using the Chiron RIBA Processor System (Chiron Corporation, Inc.), and HCV RNA was detected by reverse transcriptase-PCR amplification of the 5’ noncoding region. Individuals with either positive anti-HCV antibodies or detectable HCV RNA were classified as the HCV-infected population, while those who tested negative for anti-HCV antibodies were defined as the non-HCV population.

### Outcome

The outcomes of interest, including all-cause mortality and mortality from cardiovascular disease (CVD) or cancer, were assessed using NHANES data linked to death records from the National Death Index (NDI). Survival status was determined through probabilistic matching and death certificate review, utilizing the International Classification of Diseases, Tenth Revision (ICD-10), to classify the cause of death. Deaths were identified based on the leading causes of death listed in the publicly available NHANES linked mortality file. Death from cardiovascular disease (CVD) was defined by ICD-10 codes I00-I09, I11, I13, and I20-I51, while death from cancer was defined by codes C00-C97. Participants were followed up from the NHANES baseline interview date to either the date of their death or the last date of follow-up (December 31, 2019), whichever came first.

### Covariates

We categorized race/ethnicity based on participants’ self-reports into four groups: non-Hispanic White, non-Hispanic Black, Hispanic, and other (including multi-racial). The family income-to-poverty ratio was calculated by dividing the total family income by the poverty threshold specific to the family’s size, year, and state. Marital status was dichotomized into non-single (married or living with partners) and single (widowed, divorced, separated, or never married). Diabetes and hypertension were defined as either a self-reported diagnosis of the condition or current use of medications for these diseases. A history of cardiovascular disease was determined by self-reported diagnoses of congestive heart failure, coronary heart disease, angina, or heart attack. A history of stroke was identified through participants’ self-reported history of the condition. Body mass index (BMI) was computed by dividing body weight in kilograms by the square of height in meters. Data from the complete blood count panel, including hemoglobin (Hb) and platelet counts, as well as the biochemistry profile, including blood urea nitrogen (BUN), creatinine, aspartate aminotransferase (AST), alanine aminotransferase (ALT), total bilirubin, and albumin, were collected. The Fibrosis-4 (FIB-4) index was calculated using the formula: FIB-4  =  [Age (years)×AST (U/L)]/ [Platelets (10^9^/L)×√ALT (U/L)]. The estimated glomerular filtration rate (eGFR) was determined using the Chronic Kidney Disease Epidemiology Collaboration 2021 (CKD-EPI 2021) equation.

### Statistical analysis

The characteristics of the sample population were described using survey-weighted means with standard errors (SE) for continuous variables and counts with survey-weighted proportions for categorical variables. We performed a two-tailed test at a significance level of 0.05. Sample weights in NHANES are constructed to account for non-response, oversampling, and non-coverage. We incorporated sampling weights, clustering, and stratification into regression analyses to accommodate the complex sampling design. We performed weighted Kaplan-Meier method with Log-Rank test to plot the survival curves. Weighted Cox regression analysis was performed to explore the association between HCV status and mortality risk with adjustment for age, sex, race/ethnicity, BMI, smoking status, marital status, family income to poverty ratio, diabetes, hypertension, CVD, stroke, and survey cycle (as continuous variable). Variable selection for the Cox proportional hazards model was guided by clinical judgment, incorporating variables with significant associations as well as those with potential clinical importance despite not being significant. Data were presented as hazard ratio (HR) and 95% confidence interval (CI). Statistical computation was performed using SAS 9.4.

## Results

Among the study population, there were 604 men and 341 women with HCV infection, corresponding to weighted prevalences of 2.4% and 1.3%, respectively (Table 1 and S1 Table). The weighted prevalences of HCV viremia were 1.6% (n = 406) in men and 0.7% (n = 199) in women. The HCV-infected population, both male and female, tended to be older, more likely to be non-Hispanic Black, single (never married, widowed, divorced, or separated), and had lower family income-to-poverty ratios. Additionally, we observed that individuals with HCV infection had lower BMI, higher prevalences of hypertension, cardiovascular disease, and previous stroke, and were more likely to be current smokers. Furthermore, their blood levels of AST and ALT were higher, while albumin levels were lower compared to non-infected individuals. The FIB-4 index was also higher in the HCV-infected population.

**Table 1:** Baseline characteristics of the study population.

	Non-HCV	HCV	*p* value
No. of participants	N = 44637 (98.1%)	N = 945 (1.9%)	
Age (years old)	45.5±0.2	49.5±0.5	< 0.001
Age group (years old)			< 0.001
≤ 39	16594 (39.2%)	140 (17.0%)	
40–59	15071 (39.0%)	539 (66.6%)	
≥ 60	12972 (21.8%)	266 (16.3%)	
Sex			< 0.001
Male	21374 (48.3%)	604 (62.9%)	
Female	23263 (51.7%)	341 (37.1%)	
Race			< 0.001
White	380 (68.1%)	380 (67.8%)	
Black	320 (10.8%)	320 (17.5%)	
Hispanics	200 (14.3%)	200 (10.5%)	
Others	45 (6.8%)	45 (4.1%)	
Educational level			< 0.001
≤ high school	21923 (40.3%)	614 (61.6%)	
≥ some college	22671 (59.7%)	330 (38.4%)	
Marital status ^#^			< 0.001
Non-single	27465 (65.2%)	456 (52.4%)	
Single	16741 (34.8%)	481 (47.6%)	
Income-poverty ratio	3.04±0.03	2.08±0.08	< 0.001
Category by income-poverty ratio			< 0.001
< 1.3	12479 (20.7%)	454 (42.9%)	
1.3 to < 3.5	15248 (35.2%)	303 (34.9%)	
≥ 3.5	13128 (44.0%)	126 (22.2%)	
BMI (kg/m^2^)	28.9±0.1	27.8±0.3	<0.001
BMI group (kg/m^2^)			< 0.001
< 25	12695 (30.6%)	311 (36.5%)	
25 to < 30	14734 (33.2%)	336 (34.9%)	
≥ 30	16599 (36.2%)	276 (28.6%)	
SBP	121.7±0.2	124.4±0.8	< 0.01
DBP	71.5±0.1	74.2±0.6	< 0.001
Diabetes	5444 (8.9%)	139 (10.5%)	0.20
Hypertension	14424 (28.8%)	439 (41.9%)	< 0.001
CVD	3217 (5.9%)	108 (9.5%)	< 0.001
Stroke	1345 (2.2%)	59 (4.6%)	< 0.001
Smoking status			< 0.001
Never	24569 (54.3%)	177 (16.7%)	
Former	10520 (24.2%)	237 (24.2%)	
Current	9513 (21.5%)	529 (59.1%)	
BUN	13.3±0.1	12.6±0.2	< 0.01
Creatinine	0.870±0.002	0.871±0.010	0.91
eGFR	98.3±0.2	97.5±0.7	0.30
AST (IU/L)	24.7±0.1	47.4±1.9	< 0.001
ALT (IU/L)	25.3±0.1	50.5±2.3	< 0.001
T-Bili (mg/dL)	0.674±0.004	0.701±0.019	0.14
Hb	14.33±0.02	14.66±0.08	< 0.001
Platelet (10^9^/L)	255.2±0.7	237.5±3.6	< 0.001
Albumin (g/dL)	4.284±0.004	4.144±0.017	< 0.001
Albumin < 3.5 g/dL	1176 (1.6%)	56 (4.5%)	< 0.001
FIB-4 index	0.99±0.01	1.85±0.12	< 0.001
FIB-4 category			< 0.001
< 1.45	35608 (83.9%)	527 (63.2%)	
1.45~3.25	8102 (15.4%)	288 (25.8%)	
> 3.25	455 (0.7%)	117 (11.0%)	

AST: aspartate aminotransferase; ALT: alanine transaminase; BMI: body mass index; BUN: blood urea nitrogen; eGFR: estimated glomerular filtration rate; FIB-4 index: fibrosis-4 index; Hb: hemoglobin; HCV: hepatitis C virus; T-Bili: total bilirubin.

^#^ Non-single: married or living with partner; single: widowed/divorced/separate/never married.

**Fig 1:**
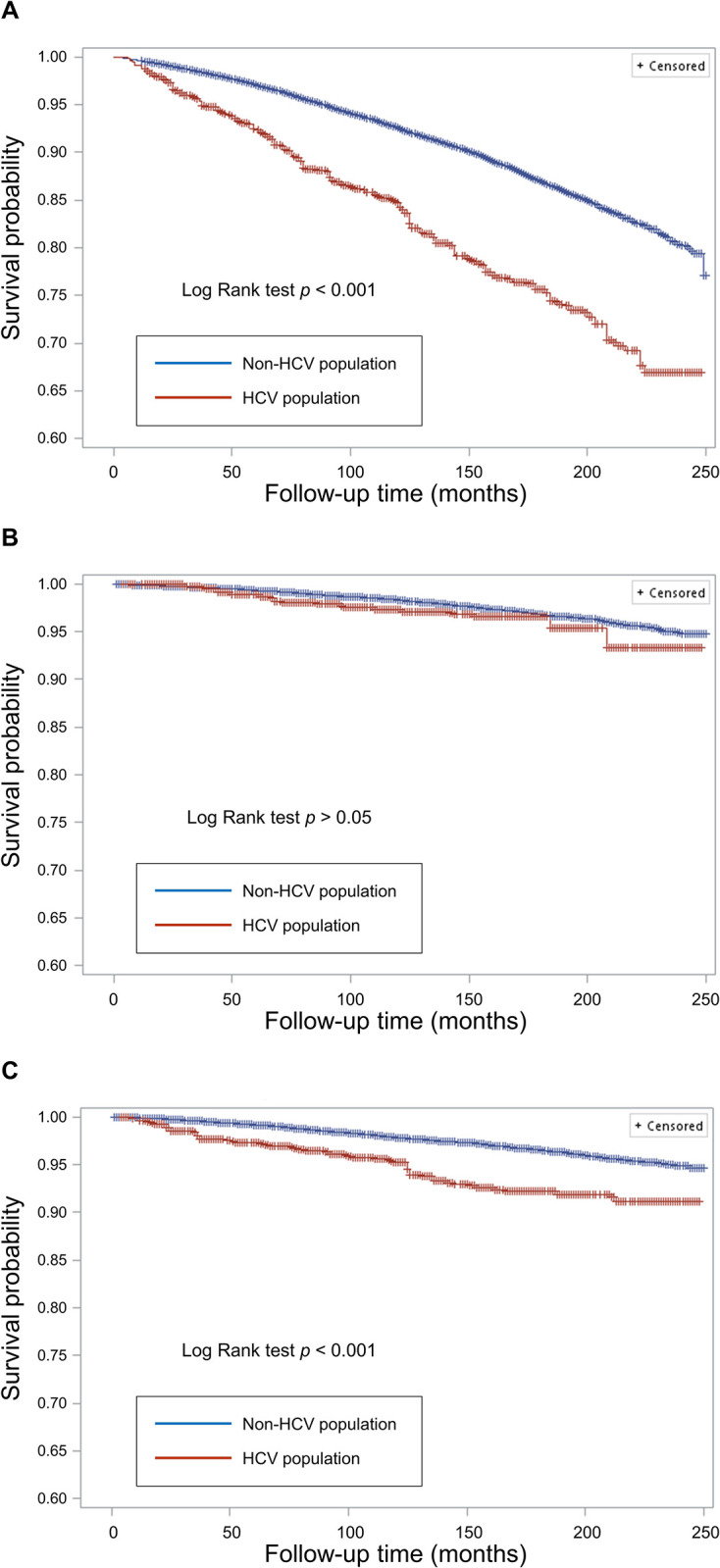
Survival curves for (A) all-cause mortality, (B) cardiovascular disease (CVD)-related mortality, and (C) cancer-related mortality, using the weighted Kaplan-Meier method by hepatitis C virus (HCV) infection status among the total population.

During a median follow-up of 125.0 months (interquartile range: 71.0–185.0 months), a total of 5,309 participants died (9.7 per 10,000 person-months), including 1,253 deaths from cardiovascular disease (CVD) and 1,319 deaths from cancer. Our results indicated that the risk of death from all causes and from cancer was higher in the HCV-infected population (Fig 1A and 1C). However, there was no difference in CVD-related mortality between the HCV-infected and non-HCV-infected populations (Fig 1B). These findings were consistent across both men (S1A–S1C Fig) and women (S2A–S2C Fig). After adjusting for age, sex, and race/ethnicity, we found that both men and women with HCV infection had a similar increased risk of death from all causes [HR 2.30 (1.79–2.96) vs. 3.07 (2.12–4.45), p for interaction > 0.05] and from cancer [HR 2.70 (1.98–3.70) vs. 3.79 (2.42–5.92), p for interaction > 0.05] compared to those without HCV infection (Table 2). After further adjusting for BMI, smoking status, marital status, family income to poverty ratio, diabetes, hypertension, CVD, previous stroke, and survey cycles, we demonstrated that both HCV-infected men (HR 1.41, 95% CI 1.10–1.81) and women (HR 2.03, 95% CI 1.36–3.02) were equally at increased risk of all-cause mortality compared to their non-HCV infected counterparts (p for interaction > 0.05). The risk of cancer-related mortality was significantly increased in HCV-infected women compared to non-HCV-infected women (HR 2.14, 95% CI 1.01–4.53). However, the association between HCV infection and cancer-related mortality among men was not statistically significant (HR 1.58, 95% CI 0.97–2.58) (Table 2).

**Table 2:** Risk of death from all causes, cardiovascular disease (CVD), and cancer in individuals with hepatitis C virus (HCV) infection compared to those without HCV infection (reference group), both for the total population and stratified by sex.

	Model 1	Model 2	Model 3
	HR (95% CI)	HR (95% CI)	HR (95% CI)
All-cause mortality			
Total population	2.55 (2.08–3.13) ***	1.60 (1.29–2.00) ***	1.58 (1.26–1.99) ***
Men	2.30 (1.79–2.96) ***	1.44 (1.12–1.85) **	1.41 (1.10–1.81) **
Women	3.07 (2.12–4.45) ***	1.99 (1.34–2.96) ***	2.03 (1.36–3.02) ***
*P* for interaction^§^	> 0.05	> 0.05	> 0.05
CVD mortality			
Total population	1.80 (1.21–2.67) **	1.16 (0.76–1.78)	1.14 (0.75–1.74)
Men	1.74 (0.89–3.42)	1.03 (0.55–1.94)	0.98 (0.53–1.83)
Women	1.83 (0.64–5.29)	1.57 (0.57–4.31)	1.67 (0.61–4.55)
*P* for interaction^§^	> 0.05	> 0.05	> 0.05
Cancer mortality			
Total population	2.70 (1.98–3.70) ***	1.70 (1.12–2.59) *	1.71 (1.12–2.60) *
Men	2.36 (1.54–3.61) ***	1.56 (0.96–2.55)	1.58 (0.97–2.58)
Women	3.79 (2.42–5.92) ***	2.15 (1.02–4.52) *	2.14 (1.01–4.53) *
*P* for interaction^§^	> 0.05	> 0.05	> 0.05

The statistical analysis used the Cox proportional hazards model, adjusting for potential confounders as follows

Model 1: adjusted for age, sex, and race

Model 2: adjusted for age, sex, race, body mass index, smoking status, marital status, and family income to poverty ratio

Model 3: adjusted for age, sex, race, body mass index, smoking status, marital status, family income to poverty ratio, diabetes, hypertension, cardiovascular disease, stroke, and survey year (as continuous variable)

*: p<0.05

**: p<0.01

***: p<0.001.

^§^: interaction between sex and HCV infection status

HR: hazard ratio; CI: confidence interval.

Among the total population, we observed a higher risk of death from all causes, CVD, and cancer in men compared to women (Table 3). These results were consistent among the non-HCV-infected population. However, among individuals infected with HCV, our results indicated no difference in the risks of all-cause mortality, CVD-related mortality, or cancer-related mortality between men and women (Table 3). When dividing the study population into four groups based on sex and HCV infection status, we observed no difference in the risk of death between HCV-infected women and non-HCV-infected men (S2 Table).

**Table 3:** Risk of death from all causes, cardiovascular disease (CVD), and cancer in men compared to women (reference group), both for the total population and stratified by hepatitis C virus (HCV) infection status.

	Model 1	Model 2	Model 3
	HR (95% CI)	HR (95% CI)	HR (95% CI)
All-cause mortality			
Total population	1.54 (1.45–1.63) ***	1.67 (1.55–1.79) ***	1.51 (1.41–1.63) ***
HCV population	1.14 (0.77–1.67)	1.32 (0.85–2.05)	1.14 (0.73–1.78)
Non-HCV population	1.53 (1.44–1.62) ***	1.66 (1.54–1.79) ***	1.52 (1.41–1.64) ***
CVD mortality			
Total population	1.77 (1.56–2.01) ***	2.14 (1.81–2.52) ***	1.90 (1.60–2.26) ***
HCV population	1.50 (0.40–5.65)	1.23 (0.33–4.65)	1.05 (0.37–2.97)
Non-HCV population	1.76 (1.55–2.01) ***	2.16 (1.82–2.56) ***	1.92 (1.61–2.28) ***
Cancer mortality			
Total population	1.78 (1.55–2.04) ***	1.70 (1.45–1.98) ***	1.55 (1.32–1.82) ***
HCV population	1.01 (0.56–1.80)	1.17 (0.53–2.60)	0.98 (0.41–2.35)
Non-HCV population	1.79 (1.55–2.07) ***	1.69 (1.44–1.98) ***	1.56 (1.33–1.83) ***

The statistical analysis used the Cox proportional hazards model, adjusting for potential confounders as follows

Model 1: adjusted for age and race

Model 2: adjusted for age, race, body mass index, smoking status, marital status, and family income to poverty ratio

Model 3: adjusted for age, race, body mass index, smoking status, marital status, family income to poverty ratio, diabetes, hypertension, cardiovascular disease, stroke, survey year (as continuous variable), and FIB-4 index (log transformed)

***: p<0.001.

HR: hazard ratio; CI: confidence interval.

## Discussion

Although HCV infection impacts health outcomes differently in men and women, there is limited research focusing on sex disparities in mortality risk. Based on a large representative sample of U.S. adults enrolled in the 1999–2018 NHANES study, with follow-up until the end of 2019, we identified an elevated risk of all-cause mortality, as well as mortality related to cancer, among individuals with HCV infection compared to those without. Our study did not show significant effect modification by sex in the relationship between HCV status and mortality risks. Additionally, among the general population, we observed a higher risk of death from all causes, CVD, and cancer in men compared to women. However, among the HCV-infected population, there were no significant differences in mortality risks between men and women.

Previous research has indicated that people with HCV infection have a higher risk of mortality compared with the general population [16, 17]. However, evidence regarding sex differences in HCV-related mortality remains inconsistent. A population-based cohort study conducted in England suggested that individuals diagnosed with HCV infection had more than double the mortality rates compared to the general population, with higher rates observed in men than in women [16]. Similarly, an Italian study reported that men with HCV infection had a higher risk of death from non-liver-related causes compared with women. Additionally, men showed a borderline significantly higher risk of death from all causes, while liver-related mortality did not differ significantly between sexes [17]. A study conducted in England using the Trent Hepatitis C cohort also suggested that there was no difference in liver-related mortality risk between sexes [18]. On the other hand, a study in the U.S. indicated that individuals with chronic HCV had more than twice the all-cause mortality rate compared to non-HCV infected individuals, with rates being even higher in women than in men. However, the study did not find statistical significance in the mortality ratio by HCV status to suggest any effect modification [19]. Our study suggested that both men and women with HCV infection experienced an increased risk of death compared to non-HCV infected counterparts. Interestingly, women with HCV infection had lower risk of developing decompensated cirrhosis and were less like to have malignant liver tumors [20]. Although we observed higher mortality risks in women, the multiplicative interaction did not reach statistical significance. Among the general population, men were associated with higher mortality risk compared with women [18], as demonstrated in our study. However, this survival advantage was attenuated in the HCV-infected population. This may indicate a health disparity in HCV care between sexes.

The insulin resistance associated with HCV infection leads to hyperglycemia, steatosis, endothelial dysfunction, and low-grade systemic inflammation, all of which contribute to vessel damage and the formation of unstable plaques [21–23]. A meta-analysis involving 68,365 participants showed an association between HCV infection and cardiovascular death, with an odds ratio of 1.65 (95% confidence interval: 1.07–2.56) [24]. However, while our study found that HCV infection was associated with prevalent hypertension, CVD, and stroke, we did not observe an association between HCV infection and prevalent diabetes or long-term CVD mortality. Other studies have shown that resolved HCV is not associated with an increased risk of chronic illness and that treatment for HCV infection is linked to a reduced risk of cardiovascular disease events [25]. The lack of association between HCV infection and CVD mortality in our study may be due to some individuals not having viremia, either because of spontaneous viral clearance or receiving antiviral treatment. In addition, our study showed that chronic HCV infection correlated to cancer mortality. Our findings were consistent with previous studies showing that chronic HCV infection is associated with higher incidence of hepatic and extrahepatic cancers and cancer-related mortality among persons with chronic hepatitis C infection [26, 27].

This study focuses on the differences in mortality risk between HCV-infected men and women, which has significant implications for public health strategies, gender-specific care, and healthcare planning. First, our findings contribute to understanding the overall burden of HCV infection on mortality rates, informing public health policies and interventions aimed at preventing, diagnosing, and treating HCV infection, particularly in high-risk populations. Second, by exploring gender disparities in HCV-related mortality, the study highlights the differential impact on men and women. This knowledge can guide healthcare providers in developing gender-specific treatment approaches and interventions to address the unique risks and needs of each gender. Third, understanding the increased risk of mortality, especially related to liver diseases and cancers, emphasizes the importance of early detection, timely management, and monitoring of HCV infection to mitigate its long-term adverse health consequences.

There were still some limitations to our study. First, our study was an observational cohort study, and the study design did not allow the establishment of a causal relationship between HCV infection and mortality outcomes. Additionally, other unmeasured variables could influence the observed associations. Second, this study’s reliance on existing NHANES data may limit the depth and specificity of certain variables, which may affect the accuracy of the results. Third, while this study highlights gender differences in HCV-related mortality, it does not delve into the broader social and cultural factors that might contribute to these disparities. Fourth, because HCV infection is typically acquired earlier in life, the duration of HCV exposure among the infected participants was unknown. This limitation may have introduced bias into our study results. Fifth, due to the unavailability of detailed causes of death in the database, we were unable to examine the association between exposure variables and the risk of liver-related mortality or mortality from specific types of cancer.

## Conclusion

Men and women with HCV infection had a similarly increased risk of death compared with their non-HCV-infected counterparts. In addition, among HCV-infected individuals, there was no difference in all-cause mortality risk between men and women. While women generally have a lower mortality risk in the general population, our results suggest that HCV infection may diminish the mortality difference between men and women.

## Supporting information

S1 Fig Survival curves for **(A)** all-cause mortality, **(B)** cardiovascular disease (CVD)-related mortality, and **(C)** cancer-related mortality, using the weighted Kaplan-Meier method by hepatitis C virus (HCV) infection status among men.
(TIF)

S2 Fig Survival curves for **(A)** all-cause mortality, **(B)** cardiovascular disease (CVD)-related mortality, and **(C)** cancer-related mortality, using the weighted Kaplan-Meier method by hepatitis C virus (HCV) infection status among women.
(TIF)

S1 Table Baseline characteristics of the study population stratified by sex and hepatitis C virus (HCV) infection status. (DOCX)

S2 Table Risk of death from all causes, cardiovascular disease (CVD), and cancer among the study population by sex and hepatitis C virus (HCV) infection status. (DOCX)
