# Supplementary figures and images for: All-cause and cause-specific mortality risk among men and women with hepatitis C virus infection
Source: PLoS One. 2024 Sep 9;19(9):e0309819. doi: 10.1371/journal.pone.0309819 (PMC11383219; doi:10.1371/journal.pone.0309819)

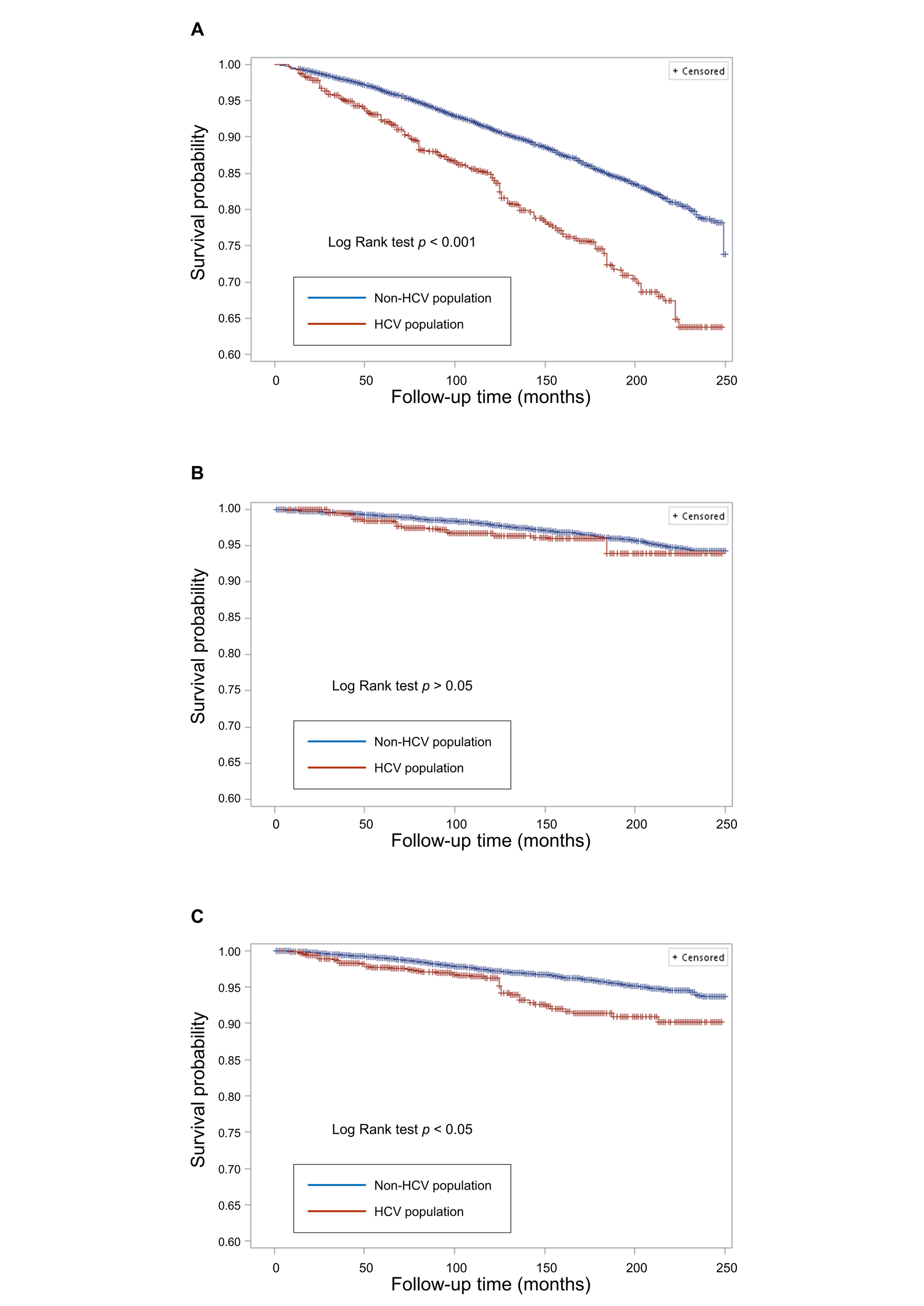

Supplement: S1 Fig — Survival curves for (A) all-cause mortality, (B) cardiovascular disease (CVD)-related mortality, and (C) cancer-related mortality, using the weighted Kaplan-Meier method by hepatitis C virus (HCV) infection status among men. (TIF) [file pone.0309819.s001.tif]

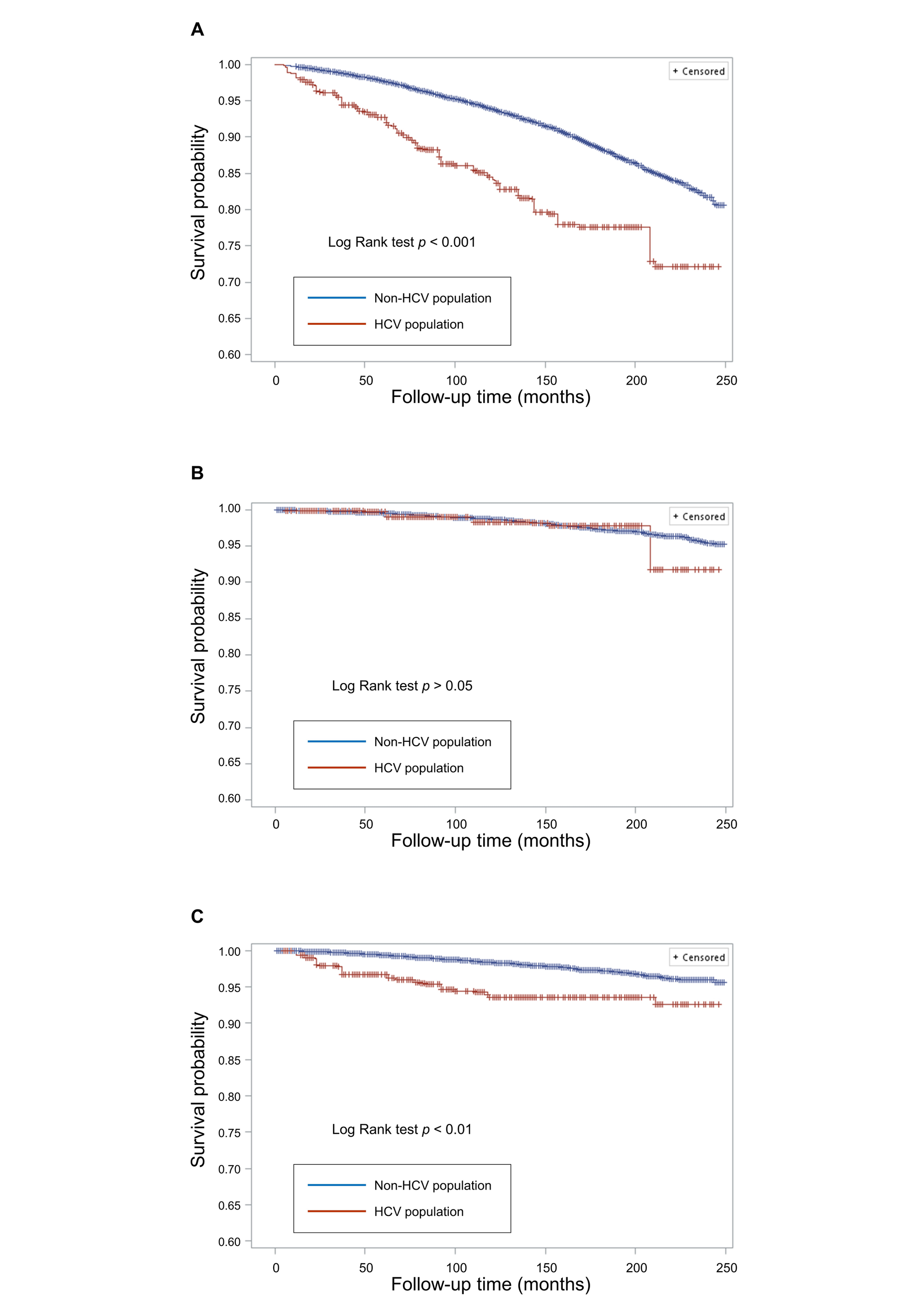

Supplement: S2 Fig — Survival curves for (A) all-cause mortality, (B) cardiovascular disease (CVD)-related mortality, and (C) cancer-related mortality, using the weighted Kaplan-Meier method by hepatitis C virus (HCV) infection status among women. (TIF) [file pone.0309819.s002.tif]
