# Supplementary material for: All-cause and cause-specific mortality risk among men and women with hepatitis C virus infection
Source: PLoS One. 2024 Sep 9;19(9):e0309819. doi: 10.1371/journal.pone.0309819 (PMC11383219; doi:10.1371/journal.pone.0309819)
Supplement: S1 Table — (DOCX) [file pone.0309819.s003.docx]

Supplemental Table S1. Baseline characteristics of the study population stratified by sex and hepatitis C virus (HCV) infection status

|  | Men | |  |  | Women | |  |
| --- | --- | --- | --- | --- | --- | --- | --- |
|  | Non-HCV | HCV | p |  | Non-HCV | HCV | p |
| No. of participants | N=21374 (97.6%) | N=604 (2.4%) |  |  | N=23263 (98.7%) | N=341 (1.3%) |  |
| Age (years) | 45.1±0.2 | 49.8±0.5 | <0.001 |  | 45.9±0.2 | 49.0±0.8 | <0.001 |
| Age group |  |  | <0.001 |  |  |  | <0.001 |
| ≤39 | 7699 (40.2%) | 77 (14.9%) |  |  | 8895 (38.3%) | 63 (20.7%) |  |
| 40-59 | 7202 (39.0%) | 352 (69.2%) |  |  | 7869 (38.9%) | 187 (62.2%) |  |
| ≥60 | 6473 (20.7%) | 175 (15.9%) |  |  | 6499 (22.8%) | 91 (17.1%) |  |
| Race |  |  | <0.001 |  |  |  | <0.001 |
| White | 9294 (68.6%) | 243 (68.0%) |  |  | 9723 (67.6) | 137 (67.5%) |  |
| Black | 4383 (9.9%) | 199 (16.5%) |  |  | 4877 (11.7%) | 121 (19.4%) |  |
| Hispanics | 5676 (14.8%) | 132 (11.1%) |  |  | 6526 (13.9%) | 68 (9.6%) |  |
| Others | 2021 (6.7%) | 30 (4.5%) |  |  | 2137 (6.8%) | 15 (3.5%) |  |
| Educational level |  |  | <0.001 |  |  |  | <0.001 |
| ≤ high school | 10922 (41.9%) | 402 (64.7%) |  |  | 11001 (38.8%) | 212 (56.4%) |  |
| ≥ some college | 10431 (58.1%) | 202 (35.3%) |  |  | 12240 (61.2%) | 128 (43.6%) |  |
| Marital status ^#^ |  |  | <0.001 |  |  |  | <0.001 |
| Non-single | 14145 (68.3%) | 315 (55.9%) |  |  | 13320 (62.3%) | 141 (46.5%) |  |
| Single | 7034 (31.7%) | 283 (44.1%) |  |  | 9707 (37.7%) | 198 (53.5%) |  |
| PIR | 3.15±0.03 | 2.18±0.09 | <0.001 |  | 2.95±0.03 | 1.91±0.16 | <0.001 |
| Category by PIR |  |  | <0.001 |  |  |  | <0.001 |
| <1.3 | 5561 (18.6%) | 279 (38.8%) |  |  | 6918 (22.8%) | 175 (49.6%) |  |
| 1.3 to < 3.5 | 7383 (34.9%) | 197 (37.7%) |  |  | 7865 (35.5%) | 106 (30.4%) |  |
| ≥ 3.5 | 6657 (46.5%) | 84 (23.5%) |  |  | 6471 (41.7%) | 42 (20.0%) |  |
| BMI (kg/m^2^) | 28.7±0.1 | 27.2±0.4 | <0.001 |  | 29.0±0.1 | 28.9±0.5 | >0.05 |
| BMI group |  |  | <0.001 |  |  |  | >0.05 |
| <25 | 5744 (26.5%) | 215 (38.2%) |  |  | 6951 (34.4%) | 96 (33.7%) |  |
| 25 to < 30 | 8147 (38.8%) | 234 (38.4%) |  |  | 6587 (28.0%) | 102 (29.0%) |  |
| ≥ 30 | 7172 (34.7%) | 140 (23.4%) |  |  | 9427 (37.6%) | 136 (37.4%) |  |
| SBP | 123.5±0.2 | 125.1±1.0 | >0.05 |  | 120.0±0.2 | 123.3±1.1 | <0.01 |
| DBP | 73.1±0.2 | 74.7±0.7 | <0.05 |  | 70.1±0.2 | 73.3±0.9 | <0.001 |
| Diabetes | 2749 (9.3%) | 89 (11.7%) | >0.05 |  | 2695 (8.5%) | 50 (8.5%) | >0.05 |
| Hypertension | 6933 (28.8%) | 263 (41.0%) | <0.001 |  | 7491 (28.8%) | 176 (43.4%) | <0.001 |
| CVD | 1913 (7.2%) | 77 (10.8%) | <0.05 |  | 1304 (4.7%) | 31 (7.1%) | >0.05 |
| Stroke | 658 (2.0%) | 35 (3.3%) | <0.05 |  | 687 (2.5%) | 24 (6.6%) | <0.01 |
| Smoking status |  |  | <0.001 |  |  |  | <0.001 |
| Never | 9693 (47.4%) | 88 (14.7%) |  |  | 14876 (60.8%) | 89 (20.0%) |  |
| Former | 6267 (28.5%) | 161 (25.4%) |  |  | 4253 (20.2%) | 76 (22.1%) |  |
| Current | 5397 (24.1%) | 353 (59.9%) |  |  | 4116 (19.0%) | 176 (57.8%) |  |
| BUN | 14.2±0.1 | 13.0±0.2 | <0.001 |  | 12.4±0.1 | 11.9±0.4 | >0.05 |
| Creatinine | 0.987±0.003 | 0.934±0.013 | <0.001 |  | 0.760±0.003 | 0.763±0.012 | >0.05 |
| eGFR | 97.8±0.2 | 98.1±0.9 | >0.05 |  | 98.8±0.3 | 96.5±1.3 | >0.05 |
| AST (IU/L) | 26.7±0.1 | 52.3±2.6 | <0.001 |  | 22.8±0.1 | 39.0±2.4 | <0.001 |
| ALT (IU/L) | 29.7±0.2 | 58.5±3.3 | <0.001 |  | 21.1±0.2 | 36.7±2.0 | <0.001 |
| T-Bili (mg/dL) | 0.761±0.005 | 0.778±0.026 | >0.05 |  | 0.593±0.003 | 0.568±0.022 | >0.05 |
| Hb | 15.24±0.02 | 15.14±0.09 | >0.05 |  | 13.47±0.02 | 13.85±0.10 | <0.001 |
| Platelet (10^9^/L) | 240.5±0.7 | 218.7±4.7 | <0.001 |  | 268.9±0.8 | 269.6±5.2 | >0.05 |
| Albumin (g/dL) | 4.392±0.005 | 4.183±0.021 | <0.001 |  | 4.183±0.005 | 4.076±0.028 | <0.001 |
| Albumin < 3.5 g/dL | 176 (0.5%) | 35 (4.5%) | <0.001 |  | 1000 (2.6%) | 21 (4.4%) | >0.05 |
| FIB-4 index | 1.03±0.01 | 2.13±0.18 | <0.001 |  | 0.95±0.01 | 1.35±0.07 | <0.001 |
| FIB-4 category |  |  | <0.001 |  |  |  | <0.001 |
| < 1.45 | 16240 (82.1%) | 16240 (55.9%) |  |  | 19368 (85.5%) | 225 (75.9%) |  |
| 1.45~3.25 | 4644 (17.0%) | 4644 (29.5%) |  |  | 3458 (13.9%) | 82 (19.3%) |  |
| > 3.25 | 280 (0.9%) | 280 (14.6%) |  |  | 175 (0.6%) | 26 (4.7%) |  |

AST: aspartate aminotransferase; ALT: alanine transaminase; BMI: body mass index; BUN: blood urea nitrogen; eGFR: estimated glomerular filtration rate; FIB-4 index: fibrosis-4 index; Hb: hemoglobin; HCV: hepatitis C virus; PIR: family income to poverty ratio; T-Bili: total bilirubin.

^#^ Non-single: married or living with partner; single: widowed/divorced/separate/never married.
