# Supplementary material for: All-cause and cause-specific mortality risk among men and women with hepatitis C virus infection
Source: PLoS One. 2024 Sep 9;19(9):e0309819. doi: 10.1371/journal.pone.0309819 (PMC11383219; doi:10.1371/journal.pone.0309819)
Supplement: S2 Table — (DOCX) [file pone.0309819.s004.docx]

Supplemental Table S2. Risk of death from all causes, cardiovascular disease (CVD), and cancer among the study population by sex and hepatitis C virus (HCV) infection status

|  | Model 1 | Model 2 | Model 3 |
| --- | --- | --- | --- |
|  | HR (95% CI) | HR (95% CI) | HR (95% CI) |
| All-cause mortality |  |  |  |
| HCV women | 1 | 1 | 1 |
| Non-HCV women | 0.34 (0.24-0.48) *** | 0.51 (0.35-0.76) *** | 0.58 (0.39-0.88) * |
| HCV men | 1.22 (0.79-1.89) | 1.26 (0.81-1.96) | 1.11 (0.72-1.71) |
| Non-HCV men | 0.51 (0.36-0.73) *** | 0.86 (0.59-1.25) | 0.89 (0.60-1.32) |
|  |  |  |  |
| CVD mortality |  |  |  |
| HCV women | 1 | 1 | 1 |
| Non-HCV women | 0.55 (0.19-1.54) | 0.64 (0.23-1.77) | 0.65 (0.23-1.81) |
| HCV men | 1.71 (0.38-7.78) | 1.41 (0.36-5.55) | 1.17 (0.30-4.56) |
| Non-HCV men | 0.96 (0.34-2.72) | 1.37 (0.49-3.81) | 1.24 (0.45-3.46) |
|  |  |  |  |
| Cancer mortality |  |  |  |
| HCV women | 1 | 1 | 1 |
| Non-HCV women | 0.26 (0.16-0.41) *** | 0.44 (0.21-0.92) * | 0.58 (0.25-1.33) |
| HCV men | 1.05 (0.58-1.91) | 1.11 (0.47-2.60) | 1.02 (0.41-2.54) |
| Non-HCV men | 0.46 (0.30-0.72) *** | 0.74 (0.35-1.60) | 0.90 (0.39-2.10) |
|  |  |  |  |

The statistical analysis used the Cox proportional hazards model, adjusting for potential confounders as follows:

Model 1: adjusted for age and race

Model 2: adjusted for age, race, body mass index, smoking status, marital status, and family income to poverty ratio

Model 3: adjusted for age, race, body mass index, smoking status, marital status, family income to poverty ratio, diabetes, hypertension, cardiovascular disease, stroke, survey year (as continuous variable), and FIB-4 index (log transformed)

^*^: p<0.05; ^***^: p<0.001.

HR: hazard ratio; CI: confidence interval.
